# CD-19 CART therapy and orthostatic hypotension: a single center retrospective cohort study

**DOI:** 10.1186/s40959-022-00132-3

**Published:** 2022-04-05

**Authors:** Ashish Patel, Joshua Levenson, Ziyu Huang, Mounzer Agha, Kathleen Dorritie

**Affiliations:** 1 Division of General Internal Medicine, University of Pittsburgh Medical Center, Pittsburgh, PA USA; 2 Division of Cardiology, UPMC Heart and Vascular Institute, University of Pittsburgh, Pittsburgh, PA USA; 3 Biostatistics Facility, UPMC Hillman Cancer Center, University of Pittsburgh, Pittsburgh, PA USA; 4 Division of Hematology and Oncology, UPMC Hillman Cancer Center, University of Pittsburgh, Pittsburgh, PA USA

**Keywords:** Chimeric antigen receptor T-cell therapy, Orthostatic hypotension, Age, Ambulatory blood pressure, Cytokine release syndrome, Hypertension

## Abstract

**Background:**

Chimeric antigen receptor T-cell (CART) therapy is a form of cellular immunotherapy used to treat hematologic malignancies. Major adverse cardiovascular events have been seen in CART patients who have high grade CRS, higher baseline creatinine, and troponin elevation. However, the incidence and factors associated with orthostatic hypotension after CART therapy have not previously been reported in the literature.

**Methods:**

We looked at patients who underwent CD-19 directed CART therapy at UPMC Shadyside hospital from April 1^st^ 2018 to December 1^st^ 2020. Patients were classified as having orthostatic hypotension if they had recorded orthostatic vital signs that were positive or provider notes indicated that vitals had been taken and were positive in the time period from discharge to 3 months post-CART. Data was analyzed with univariate and multivariate analysis using logistic regression.

**Results:**

79% of patients had orthostatic hypotension after discharge from their CART hospitalization and 64% of those patients were symptomatic. Older age, lower BMI, lower ambulatory diastolic blood pressure and grade 2 CRS were associated with orthostatic hypotension in the univariate analysis. Older age and lower ambulatory systolic blood pressure were associated with orthostatic hypotension in the multivariate analysis. Symptomatic orthostatic hypotension was associated with a history of hypertension in both the univariate and multivariate analysis. Patients with symptoms also had a higher pre-CART ejection fraction but this association was not seen in the regression model.

**Conclusion:**

There is a high incidence of orthostatic hypotension after CART therapy even after discharge. Therefore, orthostatic vitals signs and associated symptoms should be assessed in both the inpatient and outpatient setting. Older patients and patients with lower BMIs, lower ambulatory blood pressures, grade 2 CRS, or a history of hypertension may need closer monitoring.

## Introduction

There are various ways to modulate the immune system to target cancer cells. One such method involves the genetic engineering of a patient’s T cells to express an artificial T cell receptor which targets ligands expressed primarily on cancer cells [1]. Through targeting of CD 19, this chimeric antigen receptor T-cell (CART) therapy, has been shown to be effective in treating relapsed and refractory B-cell malignancies [2, 3]. While CART therapy can lead to durable remissions in a subgroup of patients, there are significant off-target adverse effects with the two most prominent being cytokine release syndrome (CRS) and neurotoxicity [4].

CRS is a systemic inflammatory process mediated by IL-6 and other cytokines that leads to signs and symptoms such as fever, hypoxia, and hypotension [5]. While most cases are mild, severe CRS can lead to multi-organ failure and death. Currently, CRS is primarily treated with tocilizumab, an antibody targeting IL-6, and glucocorticoids, both of which can help reduce the severity of CRS [6]. The underlying mechanism leading to neurotoxicity, also known as immune effector cell-associated neurotoxicity syndrome (ICANS), is less well understood and is an active area of research. [7]. Symptoms of ICANS can range from mild headache and anxiety to severe impairment including delirium, seizures, and even coma [8]. The primary approach to the treatment of neurotoxicity is glucocorticoids [9]. While CRS and ICANS are well established side effects of CART-therapy, it wasn’t until recently that cardiovascular effects of CART therapy were characterized.

Patients undergoing CART therapy can experience cardiovascular mortality, new onset heart failure, heart failure decompensation, and new arrhythmias [10, 11]. In one retrospective cohort study, cardiovascular events occurred in 12% of patients and they overwhelmingly occurred in patients who had grade 2 or higher CRS and a post-CART positive troponin [12]. In another study, cardiovascular events occurred in 21% of patients and were associated with grade 3 CRS, grade 4 CRS, and a higher baseline creatinine [13]. However, these and other previous studies have not described orthostatic hypotension in patients after CART therapy.

In this single center retrospective cohort study, we report on the incidence of orthostatic hypotension after CART therapy. We also collected data on past medical history, medications, labs, echo parameters, CRS grade, neurotoxicity, and oncologic histories to find factors associated with orthostatic hypotension.

## Methods

Our cohort consisted of patients who underwent CD-19 directed CART therapy at UPMC Shadyside hospital from April 1^st^ 2018 to December 1^st^ 2020. Patients who did not have orthostatic vital signs measured after their discharge from CART hospitalization were excluded. Patients with frank hypotension defined as a systolic blood pressure less than 90 in at least two different positions (supine, sitting, or standing) were also excluded. Diagnosis of orthostatic hypotension was made if recorded vitals met the criteria for orthostatic hypotension or if provider notes clearly stated that orthostatic vital signs were performed and positive even if the vitals themselves were not charted. Standard vitals criteria were used for orthostatic hypotension: ≥ 20 mmHg drop in systolic blood pressure or a ≥ 10 mmHg drop in diastolic blood pressure from a supine or sitting position to the standing position. Patients were placed in the orthostatic hypotension group if they had orthostasis between the time of discharge from CART hospitalization to 3 months post-CART therapy. Patients were categorized as symptomatic if provider notes indicated they were symptomatic from their orthostatic hypotension or if provider notes mentioned that patients were lightheaded or fatigued upon standing within a one-week period of their positive orthostatic vital signs.

All data collected with regards to patient characteristics, past medical history, oncologic history, pre-CART vitals, CART hospitalization, post-CART vitals, medications, CART side effects, echo parameters, and labs was obtained via manual chart review. Three sets of ambulatory vital signs from different outpatient visits prior to CART therapy for each patient were included in the study. Pre-CART baseline orthostatic vital signs were defined as any vitals taken between admission and CART infusion. If there were multiple vital signs, then those closest to the infusion were used. Discharge vitals were taken within 48 h prior to discharge and discharge labs within 24 h prior to discharge. Pre-CART labs (except pre-conditioning hemoglobin) were done within 24 h prior to CART infusion. Pre-conditioning hemoglobin was defined as the hemoglobin that was done prior to lymphodepleting chemotherapy being given on Day 1 of conditioning. All patients had at least 3 months of follow-up time for post-CART data collection as data was collected until March 1^st^ 2021. Our center uses the American Society for Transplantation and Cellular Therapy (ASTCT) consensus grading scheme to determine the severity of CRS and ICANS. Echo parameters were collected from echo reports. All oncologic treatments listed were given prior to the start of lymphodepletion.

Univariate statistical analysis was conducted using t-tests for most continuous variables and Kruskal -Wallis tests for non-normally distributed continuous variables. Chi-squared tests were used for most categorical variables with large numbers, and Fisher’s exact tests were used for ones with small counts. Multivariate statistical analysis was conducted using a logistic regression model. Variables were selected for the multivariate analysis based on the results of the univariate analysis and variables we deemed clinically relevant. For the univariate and multivariate analysis an alpha level of 0.05 was used. Statistical analysis was conducted in R version 4.1.0.

## Results

We had 56 patients that underwent CART therapy during our study period that we had access to in our electronic medical records. Out of those 56 patients, 11 patients did not have their orthostatic vital signs assessed after discharge from their CART hospitalization. 3 patients had frank hypotension. Out of the remaining 42 patients, 33 patients (79%) had orthostatic hypotension after CART discharge while 9 patients did not (Table 1). 40 patients (95.2%) received YESCARTA, while 2 patients (4.8%) received KYMRIAH (Table 1). 9 patients (21.4%) had a history of orthostatic hypotension; however, there was no statistically significant difference between patients with and without orthostatic hypotension after CART (Table 1).

**Table 1:** Characteristics of patients with orthostatic hypotension after CART hospitalization

	Cohort	No orthostatic hypotension	Orthostatic hypotension	*p*-value
n	42	9	33	
**Patient Characteristics**				
Age (mean (SD))	63.26 (14.99)	49.33 (19.64)	67.06 (11.05)	0.001
Sex (%)				0.575
Male	27 (64.3)	7 (77.8)	20 (60.6)	
Female	15 (35.7)	2 (22.2)	13 (39.4)	
Race (%)				1
White	41 (97.6)	9 (100.0)	32 (97.0)	
Black	1 (2.4)	0 (0.0)	1 (3.0)	
BMI (mean (SD))	28.83 (5.80)	32.24 (6.76)	27.90 (5.25)	0.045
**Past Medical History**				
Hypertension (%)	21 (50.0)	4 (44.4)	17 (51.5)	1
Hyperlipidemia (%)	19 (45.2)	1 (11.1)	18 (54.5)	0.052
Type 2 Diabetes Mellitus (%)	5 (11.9)	2 (22.2)	3 (9.1)	0.619
Coronary artery disease (%)	2 (4.8)	1 (11.1)	1 (3.0)	0.9
Chronic kidney disease (%)	8 (19.0)	0 (0.0)	8 (24.2)	0.245
Myocardial infarction (%)	1 (2.4)	0 (0.0)	1 (3.0)	1
Atrial Fibrillation (%)	2 (4.8)	0 (0.0)	2 (6.1)	1
Tobacco Use (%)	24 (57.1)	4 (44.4)	20 (60.6)	0.625
Orthostatic hypotension (%)	9 (21.4)	2 (22.2)	7 (21.2)	1
Neuropathy (%)	26 (61.9)	5 (55.6)	21 (63.6)	0.711
**Oncologic History**				
Cancer type (%)				
ALL	2 (4.8)	2 (22.2)	0 (0.0)	
DLBCL	39 (92.9)	6 (66.7)	33 (100.0)	
DLBCL/CHL	1 (2.4)	1 (11.1)	0 (0.0)	
CAR-T Product (%)				0.058
YESCARTA	40 (95.2)	7 (77.8)	33 (100.0)	
KYMRIAH	2 (4.8)	2 (22.2)	0 (0.0)	
Chest/axillary radiation (%)	10 (23.8)	1 (11.1)	9 (27.3)	0.57
Neck radiation (%)	6 (14.3)	1 (11.1)	5 (15.2)	1
Stem cell transplant (%)	9 (21.4)	2 (22.2)	7 (21.2)	1
R-CHOP (%)	27 (64.3)	3 (33.3)	24 (72.7)	0.073
R-ICE (%)	18 (42.9)	3 (33.3)	15 (45.5)	0.786
GEM-OX (%)	13 (31.0)	2 (22.2)	11 (33.3)	0.816
R-EPOCH (%)	9 (21.4)	4 (44.4)	5 (15.2)	0.15
**Pre-CART vital signs**				
Ambulatory mean SBP (mean (SD))	119.93 (12.31)	125.30 (4.18)	118.46 (13.39)	0.142
Ambulatory mean DBP (mean (SD))	73.21 (7.45)	78.70 (2.27)	71.71 (7.68)	0.011
Ambulatory mean HR (mean (SD))	84.18 (13.23)	88.00 (12.24)	83.14 (13.48)	0.335
Pre-CART orthostatic vital signs assessed (%)	16 (38.1)	5 (55.6)	11 (33.3)	0.407
Pre-CART orthostatic vital signs positive (%)^a^	8 (50.0)	2 (40.0)	6 (54.5)	1
**CART Hospitalization**				
CART hospitalization time (mean (SD))	18.86 (13.71)	23.67 (24.72)	17.55 (8.93)	0.24
Time from CART infusion to discharge (mean (SD))	15.38 (9.80)	19.89 (16.72)	14.15 (6.78)	0.121
Orthostatic vitals assessed (%)	36 (85.7)	8 (88.9)	28 (84.8)	1
Orthostatic vital signs positive (%)^a^	33 (91.7)	7 (87.5)	26 (92.9)	1
**Post-CART vital signs**				
Discharge orthostatic vital signs assessed (%)	33 (78.6)	8 (88.9)	25 (75.8)	0.694
Discharge orthostatic vital signs positive (%)^a^	18 (54.5)	3 (37.5)	15 (60.0)	0.481
1 month orthostatic vital signs assessed (%)	36 (85.7)	8 (88.9)	28 (84.8)	1
1 month orthostatic vital signs positive (%)^a^	13 (36.1)	0 (0.0)	13 (46.4)	0.046
3 months orthostatic vital signs assessed (%)	13 (31.0)	2 (22.2)	11 (33.3)	0.816
3 months orthostatic vital signs positive (%)^a^	3 (23.1)	0 (0.0)	3 (27.3)	1
Discharge HR (mean (SD))	85.69 (15.43)	88.11 (14.01)	85.03 (15.93)	0.602
1 month HR (mean (SD))	84.50 (14.55)	85.50 (10.39)	84.23 (15.61)	0.83
3 months HR (mean (SD))	83.12 (13.47)	79.62 (11.10)	84.19 (14.13)	0.41
**CART side effects**				
CRS (%)	38 (90.5)	8 (88.9)	30 (90.9)	1
CRS Grade (%)				0.043
1	14 (33.3)	6 (66.7)	8 (24.2)	
2	24 (57.1)	2 (22.2)	22 (66.7)	
Tocilizumab (%)	36 (85.7)	7 (77.8)	29 (87.9)	0.818
Neurotoxicity (%)	24 (57.1)	3 (33.3)	21 (63.6)	0.212
Neurotoxicity grade (%)				0.177
1	6 (14.3)	2 (22.2)	4 (12.1)	
2	11 (26.2)	1 (11.1)	10 (30.3)	
3	7 (16.7)	0 (0.0)	7 (21.2)	
Steroids (%)	25 (59.5)	3 (33.3)	22 (66.7)	0.155
**Echo parameters**				
Pre-CART ejection fraction (mean (SD)) *n* = 37	54.68 (5.51)	53.11 (4.83)	55.18 (5.70)	0.334
Pre-CART global longitudinal strain (mean (SD)) *n* = 18	17.21 (3.10)	-14.60 (2.69)	-17.73 (2.98)	0.112
Post-CART ejection fraction (mean (SD)) *n* = 25	53.44 (6.51)	54.00 (5.81)	53.18 (6.98)	0.775
Post-CART global longitudinal strain (mean (SD)) *n* = 14	16.52 (3.25)	-16.37 (0.59)	-16.56 (3.69)	0.93
**Labs**				
Positive troponin (%)	1 (2.4)	0 (0.0)	1 (3.0)	0.558
Negative troponin (%)	9 (21.4)	3 (33.3)	6 (18.2)	
Troponin not measured (%)	32 (76.2)	6 (66.7)	26 (78.8)	
Pre-CART creatinine (mean (SD))	0.97 (0.52)	0.90 (0.32)	0.99 (0.57)	0.637
Peak creatinine (mean (SD))	1.24 (0.62)	1.31 (0.58)	1.22 (0.63)	0.703
Pre-CART HsCRP (mean (SD))	7.02 (6.59)	5.01 (4.57)	7.56 (7.00)	0.309
Peak HsCRP (mean (SD))	13.96 (8.50)	12.53 (8.80)	14.36 (8.51)	0.574
Pre-conditioning hemoglobin (mean (SD))	10.10 (1.58)	10.72 (2.56)	9.93 (1.19)	0.187
CART discharge hemoglobin (mean (SD))	9.60 (1.09)	9.72 (1.16)	9.57 (1.09)	0.72

*ALL* Acute Lymphocytic Leukemia, *DLBCL* Diffuse Large B-Cell Lymphoma, *CHL* Classical Hodgkin Lymphoma, *R-CHOP* Rituximab, Cyclophosphamide, Doxorubicin, Vincristine, Prednisone, *R-ICE* Rituximab, Ifosfamide, Carboplatin, Etoposide, *GEM-OX* Gemcitabine, Oxaliplatin, *R-EPOCH* Rituximab, Etoposide, Prednisone, Vincristine, Cyclophosphamide, Doxorubicin, *SBP* Systolic blood pressure, *DBP* Diastolic blood pressure; *HR* Heart rate, *CRS* Cytokine release syndrome, *HsCRP* High sensitivity C- reactive protein, *CART* Chimeric antigen receptor T-cell

Bolded text represents subheadings

^a^Percentage based on the number of patients who had orthostatic vital signs assessed

Patients with orthostatic hypotension had a higher mean age of 67.06 ± 11.05 years compared to 49.33 ± 19.64 years (*p* = 0.001) for patients without orthostatic hypotension (Table 1). Patients with orthostatic hypotension had a lower mean body mass index (BMI), 27.90 ± 5.25 vs. 32.24 ± 6.76 (*p* = 0.045) (Table 1). Patients with orthostatic hypotension had a lower mean ambulatory diastolic blood pressure of 71.71 ± 7.68 mmHg compared to 78.70 ± 2.27 mmHg (*p* = 0.011) for patients without orthostatic hypotension. Orthostatic patients were more likely to have Grade 2 CRS (*p* = 0.043) (Table 1).

**Table 2:** Medications in patients with orthostatic hypotension and CART therapy

	Cohort (%)	No orthostatic hypotension (%)	Orthostatic hypotension (%)	*p*-value
n	42	9	33	
**Pre-CART**				
Beta blocker	7 (16.7)	2 (22.2)	5 (15.2)	0.631
ACEI/ARB	6 (14.3)	2 (22.2)	4 (12.1)	0.593
CCB	4 (9.5)	0 (0.0)	4 (12.1)	0.561
Thiazide diuretic	3 (7.1)	1 (11.1)	2 (6.1)	0.525
Loop diuretic	2 (4.8)	1 (11.1)	1 (3.0)	0.387
Arterial vasodilator	1 (2.4)	0 (0.0)	1 (3.0)	1
Anti-depressants	6 (14.3)	1 (11.1)	5 (15.2)	1
Neuroleptics	4 (9.5)	1 (11.1)	3 (9.1)	1
Alpha-1 blockers	1 (2.4)	0 (0.0)	1 (3.0)	1
Midodrine	0 (0.0)	0 (0.0)	0 (0.0)	1
Fludrocortisone	1 (2.4)	0 (0.0)	1 (3.0)	1
**CART discharge**				
Beta blocker	9 (21.4)	3 (33.3)	6 (18.2)	0.375
ACEI/ARB	2 (4.8)	0 (0.0)	2 (6.1)	1
CCB	4 (9.5)	0 (0.0)	4 (12.1)	0.561
Thiazide diuretic	1 (2.4)	0 (0.0)	1 (3.0)	1
Loop diuretic	1 (2.4)	0 (0.0)	1 (3.0)	1
Arterial vasodilator	1 (2.4)	0 (0.0)	1 (3.0)	1
Anti-depressants	10 (23.8)	1 (11.1)	9 (27.3)	0.416
Neuroleptics	2 (4.8)	1 (11.1)	1 (3.0)	0.387
Alpha-1 blockers	4 (9.5)	0 (0.0)	4 (12.1)	0.561
Midodrine	2 (4.8)	0 (0.0)	2 (6.1)	1
Fludrocortisone	2 (4.8)	0 (0.0)	2 (6.1)	1
**1 month post-CART**				
Beta blocker	6 (14.3)	2 (22.2)	4 (12.1)	0.593
ACEI/ARB	1 (2.4)	0 (0.0)	1 (3.0)	1
CCB	3 (7.1)	0 (0.0)	3 (9.1)	1
Thiazide diuretic	0 (0.0)	0 (0.0)	0 (0.0)	1
Loop diuretic	1 (2.4)	0 (0.0)	1 (3.0)	1
Arterial vasodilator	0 (0.0)	0 (0.0)	0 (0.0)	1
Anti-depressants	9 (21.4)	1 (11.1)	8 (24.2)	0.655
Neuroleptics	3 (7.1)	1 (11.1)	2 (6.1)	0.525
Alpha-1 blockers	4 (9.5)	0 (0.0)	4 (12.1)	0.561
Midodrine	6 (14.3)	0 (0.0)	6 (18.2)	0.312
Fludrocortisone	5 (11.9)	0 (0.0)	5 (15.2)	0.567
**3 months post-CART**				
Beta blocker	7 (16.7)	3 (33.3)	4 (12.1)	0.155
ACEI/ARB	2 (4.8)	0 (0.0)	2 (6.1)	1
CCB	2 (4.8)	0 (0.0)	2 (6.1)	1
Thiazide diuretic	0 (0.0)	0 (0.0)	0 (0.0)	1
Loop diuretic	1 (2.4)	0 (0.0)	1 (3.0)	1
Arterial vasodilator	0 (0.0)	0 (0.0)	0 (0.0)	1
Anti-depressants	10 (23.8)	1 (11.1)	9 (27.3)	0.416
Neuroleptics	3 (7.1)	0 (0.0)	3 (9.1)	1
Alpha-1 blockers	1 (2.4)	0 (0.0)	1 (3.0)	1
Midodrine	4 (9.5)	0 (0.0)	4 (12.1)	0.561
Fludrocortisone	7 (16.7)	0 (0.0)	7 (21.2)	0.314

*ACEI* ACE inhibitor, *ARB* Angiotensin receptor blocker, *CART* Chimeric antigen receptor T-cell, *CCB* Calcium channel blocker

Bolded text represents subheadings

A minority of patients with orthostatic hypotension were prescribed fludrocortisone or midodrine (Table 2). In patients with orthostatic hypotension, beta blockers and anti-depressants were the most common medications prescribed prior to CART (Table 2). After CART, anti-depressants alone were the most common medication class (Table 2). There were no statistically significant differences in the usage of any orthostasis related medication classes between patients with and without orthostatic hypotension (Table 2). In addition, there was no statistically significant difference in the number of medications classes patients were discharged on from their CART hospitalization (Table 3).

**Table 3:** Orthostasis related medications and CART hospitalization discharge

	No orthostatic hypotension	Orthostatic hypotension	*p*-value
n	9	33	
Number of medication classes at discharge^a^			0.685
0	5 (55.6)	11 (33.3)	
1	3 (33.3)	16 (48.5)	
2	1 (11.1)	5 (15.2)	
3	0 (0.0)	1 (3.0)	

^a^Included medication classes: Beta blocker, ACEI/ARB, CCB, Thiazide diuretic, Loop diuretic, Arterial vasodilator, Anti-depressants, Neuroleptics, and Alpha-1 blockers

Out of 33 patients with orthostatic hypotension, 21 patients (64%) were symptomatic while 12 patients were asymptomatic (Table 4). A majority of patients (61.9%) had lightheadedness and fatigue (Table 4). Patients who were symptomatic were more likely to have a history of hypertension (*p* = 0.008) (Table 4). Symptomatic patients also had a shorter CART hospitalization time of 15.10 ± 5.48 days compared to 21.83 ± 12.07 days (*p* = 0.035) for asymptomatic patients (Table 4). Symptomatic patients also had a higher pre-CART ejection fraction than asymptomatic patients, 57.06 ± 5.62% vs. 52.27 ± 4.67% (*p* = 0.027) (Table 4).

**Table 4:** Characteristics of symptomatic orthostatic patients after CART hospitalization

	Asymptomatic	Symptomatic	*p*-value
n	12	21	
**Symptoms**			
Lightheadedness only (%)		3 (14.3)	
Fatigue only (%)		5 (23.8)	
Lightheadedness and Fatigue (%)		13 (61.9)	
**Patient Characteristics**			
Age (mean (SD))	63.00 (14.47)	69.38 (8.03)	0.112
Sex (%)			0.866
Male	8 (66.7)	12 (57.1)	
Female	4 (33.3)	9 (42.9)	
Race (%)			1
White	12 (100.0)	20 (95.2)	
Black	0 (0.0)	1 (4.8)	
BMI (mean (SD))	26.80 (5.40)	28.53 (5.18)	0.371
**Past Medical History**			
Hypertension (%)	2 (16.7)	15 (71.4)	0.008
Hyperlipidemia (%)	5 (41.7)	13 (61.9)	0.447
Type 2 Diabetes Mellitus (%)	1 (8.3)	2 (9.5)	1
Coronary artery disease (%)	0 (0.0)	1 (4.8)	1
Chronic kidney disease (%)	3 (25.0)	5 (23.8)	1
Myocardial infarction (%)	0 (0.0)	1 (4.8)	1
Atrial Fibrillation (%)	0 (0.0)	2 (9.5)	0.73
Tobacco Use (%)	8 (66.7)	12 (57.1)	0.866
Orthostatic hypotension (%)	3 (25.0)	4 (19.0)	1
Neuropathy (%)	7 (58.3)	14 (66.7)	0.716
**Oncologic Treatment**			
CAR-T Product (%)			
YESCARTA	12 (100.0)	21 (100.0)	
KYMRIAH	0 (0.0)	0 (0.0)	
Chest/axillary radiation (%)	4 (33.3)	5 (23.8)	0.853
Neck radiation (%)	1 (8.3)	4 (19.0)	0.63
Stem cell transplant (%)	2 (16.7)	5 (23.8)	0.968
R-CHOP (%)	9 (75.0)	15 (71.4)	1
R-ICE (%)	5 (41.7)	10 (47.6)	1
GEM-OX (%)	5 (41.7)	6 (28.6)	0.701
R-EPOCH (%)	2 (16.7)	3 (14.3)	1
**Pre-CART vital signs**			
Ambulatory mean SBP (mean (SD))	114.83 (14.80)	120.54 (12.42)	0.245
Ambulatory mean DBP (mean (SD))	71.78 (9.80)	71.67 (6.45)	0.969
Ambulatory mean HR (mean (SD))	82.19 (14.91)	83.68 (12.95)	0.766
Pre-CART orthostatic vital signs assessed (%)	4 (33.3)	7 (33.3)	1
Pre-CART orthostatic vital signs positive (%)^a^	3 (75.0)	3 (42.9)	0.689
**CART Hospitalization**			
C ART hospitalization time (mean (SD))	21.83 (12.07)	15.10 (5.48)	0.035
Time from CART infusion to discharge (mean (SD))	15.67 (9.01)	13.29 (5.17)	0.34
Orthostatic vitals assessed (%)	10 (83.3)	18 (85.7)	1
Orthostatic vital signs positive (%)^a^	10 (100.0)	16 (88.9)	0.743
**Post-CART vital signs**			
Discharge orthostatic vital signs assessed (%)	9 (75.0)	16 (76.2)	1
Discharge orthostatic vital signs positive (%)^a^	5 (55.6)	10 (62.5)	1
1 month orthostatic vital signs assessed (%)	11 (91.7)	17 (81.0)	0.748
1 month orthostatic vital signs positive (%)^a^	3 (27.3)	10 (58.8)	0.212
3 months orthostatic vital signs assessed (%)	5 (41.7)	6 (28.6)	0.701
3 months orthostatic vital signs positive (%)^a^	2 (40.0)	1 (16.7)	0.853
Discharge HR (mean (SD))	79.25 (12.71)	88.33 (16.91)	0.117
1 month HR (mean (SD))	83.45 (16.46)	84.68 (15.53)	0.839
3 months HR (mean (SD))	83.36 (12.96)	84.80 (15.36)	0.804
Difference between supine/sitting and standing SBP (median [IQR])	23.50 [17.50, 32.75]	25.00 [19.00, 31.50]	0.792
Difference between supine/sitting and standing DBP (median [IQR])	10.00 [2.50, 17.25]	10.00 [4.50, 14.00]	0.745
**Medications**			
Pre-CART midodrine (%)	0 (0.0)	0 (0.0)	1
CART discharge midodrine (%)	1 (8.3)	1 (4.8)	1
1 month post CART-midodrine (%)	2 (16.7)	4 (19.0)	1
3 months post-CART midodrine (%)	1 (8.3)	3 (14.3)	1
Pre-CART fludrocortisone (%)	1 (8.3)	0 (0.0)	0.364
CART discharge fludrocortisone (%)	2 (16.7)	0 (0.0)	0.125
1 month post-CART fludrocortisone (%)	3 (25.0)	2 (9.5)	0.328
3 months post-CART fludrocortisone (%)	3 (25.0)	4 (19.0)	0.686
**CART side effects**			
CRS (%)	12 (100.0)	18 (85.7)	0.457
CRS Grade (%)			0.229
1	2 (16.7)	6 (28.6)	
2	10 (83.3)	12 (57.1)	
Tocilizumab (%)	12 (100.0)	17 (81.0)	0.29
Neurotoxicity (%)	9 (75.0)	12 (57.1)	0.516
Neurotoxicity grade (%)			0.322
1	1 (8.3)	3 (14.3)	
2	6 (50.0)	4 (19.0)	
3	2 (16.7)	5 (23.8)	
Steroids (%)	10 (83.3)	12 (57.1)	0.25
**Echo parameters**			
Pre-CART ejection fraction (mean (SD)) *n* = 28	52.27 (4.67)	57.06 (5.62)	0.027
Post-CART ejection fraction (mean (SD)) *n* = 15	49.29 (8.38)	55.90 (4.43)	0.051
**Labs**			
Positive troponin (%)	1 (8.3)	0 (0.0)	0.077
Negative troponin (%)	4 (33.3)	2 (9.5)	
No troponin measured (%)	7 (58.3)	19 (90.5)	
Pre-CART creatinine (mean (SD))	0.97 (0.57)	1.01 (0.58)	0.838
Peak creatinine (mean (SD))	1.30 (0.83)	1.18 (0.50)	0.596
Pre-CART HsCRP (mean (SD))	9.08 (8.16)	6.70 (6.28)	0.355
Peak HsCRP (mean (SD))	15.96 (9.51)	13.44 (7.99)	0.422
Pre-conditioning hemoglobin (mean (SD))	9.85 (1.12)	9.98 (1.25)	0.766
CART discharge hemoglobin (mean (SD))	9.78 (1.36)	9.46 (0.91)	0.428

*R-CHOP* Rituximab, Cyclophosphamide, Doxorubicin, Vincristine, Prednisone, *R-ICE* Rituximab, Ifosfamide, Carboplatin, Etoposide, *GEM-OX* Gemcitabine, Oxaliplatin, *R-EPOCH* Rituximab, Etoposide, Prednisone, Vincristine, Cyclophosphamide, Doxorubicin, *SBP* Systolic blood pressure, *DBP* Diastolic blood pressure, *HR* Heart rate, *CRS* Cytokine release syndrome, *HsCRP* High sensitivity C- reactive protein, *CART* Chimeric antigen receptor T-cell

Bolded text represents subheadings

^a^Percentage based on the number of patients who had orthostatic vital signs assessed

Multivariate analysis showed that older age was associated with orthostatic hypotension with an odds ratio of 1.12 (CI: 1.04 – 1.26) (Table 5). In addition, ambulatory mean systolic blood pressure had a statistically significant odds ratio of 0.88 (CI: 0.75–0.98) indicating a lower ambulatory mean systolic blood pressure was associated with orthostatic hypotension in general (Table 5). However, when symptomatic patients were analyzed separately from asymptomatic patients, a history of hypertension correlated with symptomatic orthostatic hypotension with an odds ratio of 14.27 (CI: 1.75–234.2) (Table 6). Multivariate analysis of only symptomatic patients showed that pre-CART ejection fraction and age were not associated with symptomatic orthostatic hypotension (Table 6).

**Table 5:** Multivariate associations to orthostatic hypotension after CART therapy

	Odds ratio	Confidence interval
Age	1.12	1.04—1.26
Sex	0.15	0.01—1.71
BMI	0.92	0.74—1.10
CRS Grade 1	6.42	0.13—430
CRS Grade 2	50.75	0.74—12,194
Ambulatory mean SBP	0.88	0.75—0.98

*CRS* Cytokine release syndrome, *SBP* Systolic blood pressure

**Table 6:** Multivariate analysis of symptomatic orthostatic hypotension

	Odds ratio	Confidence interval
Age	1.09	0.97—1.31
Pre-CART ejection fraction	1.18	0.98—1.59
History of hypertension	14.27	1.75—234.2
CART hospitalization time	0.99	0.81—1.16

*CART* Chimeric antigen receptor T-cell

We also analyzed our cohort with respect to CRS grade and positional changes in blood pressure. There was no association between severity of CRS and differences in supine/sitting and standing blood pressures (Table 7). Symptomatic and asymptomatic patients also had similar differences between supine/sitting and standing blood pressures (Table 4). Also, there were no statistically significant differences between midodrine and fludrocortisone use among patients with and without symptoms (Table 4).

**Table 7:** CRS and differences in sitting/supine and standing blood pressures

	No CRS	CRS Grade 1	CRS Grade 2	*p*-value
n	4	14	24	
Difference between supine/sitting and standing SBP (median [IQR])	18.00 [17.00, 26.00]	31.00 [20.50, 33.50]	24.00 [20.00, 29.00]	0.686
Difference between supine/sitting and standing DBP (median [IQR])	8.00 [5.00, 10.50]	12.00 [7.00, 15.50]	10.00 [3.00, 18.00]	0.832

*CRS* Cytokine release syndrome, *SBP* Systolic blood pressure, *DBP* Diastolic blood pressure

## Discussion

In our cohort, 79% of patients had orthostatic hypotension after CART hospitalization. This is significantly higher than the general elderly population which has been reported as approximately 20% [14]. While the focus of CART toxicity is primarily neurotoxicity and CRS, orthostatic hypotension will be important to track in all CART patients as it has been shown to be associated with all-cause mortality, heart failure, and atherosclerotic cardiovascular disease in the general population [15]. In addition, patients with orthostatic hypotension have a higher risk of falls and emergency department visits for hypotension [16, 17]. It is well known that orthostatic hypotension is more prevalent in the elderly and similarly in our cohort orthostatic patients were older (*p* = 0.001) [18]. These patients had a lower BMI (*p* = 0.045) as well which has also been reported in the general population [19]. Lower ambulatory diastolic blood pressure was associated with orthostatic hypotension in the univariate analysis (*p* = 0.011) and a lower ambulatory systolic blood pressure in our multivariate analysis. However, it is important to note that both groups had close to normal ambulatory blood pressures. Orthostatic patients had more grade 2 CRS than grade 1 CRS (*p* = 0.043). A previous study has shown that higher grade CRS is associated with major adverse cardiovascular events [13]. Hypotension is one of the cardinal signs of CRS so this finding raises the question as to whether prolonged orthostatic hypotension is a sub-acute to chronic manifestation of CRS. However, it has to be acknowledged that the majority of our orthostatic patients received steroids and the vast majority received tocilizumab. In addition, CRS grade severity did not result in greater differences between sitting/supine and standing blood pressures. Therefore, CRS is unlikely to be the dominant cause of orthostatic hypotension based on our data.

Another potential cause of orthostatic hypotension we investigated is medications. Certain classes of medications like anti-hypertensives, diuretics, anti-depressants, and neuroleptics are known to cause or exacerbate orthostatic hypotension. Use of these medications trended higher numerically and percentage-wise after CART among patients in our cohort with orthostatic hypotension suggesting a potential iatrogenic etiology. However, there were no statistically significant differences in medication use between patients with and without orthostatic hypotension. We suspect that the lack of statistical significance is due to the low number of patients on these classes of medications. Larger cohorts will be needed to further investigate this potential etiology.

The majority (64%) of patients with orthostatic hypotension were also symptomatic. More than half of symptomatic patients had lightheadedness and fatigue. 23.8% of symptomatic patients had fatigue and no lightheadedness. Fatigue can be quite non-specific so it is hard to only ascribe it to orthostatic hypotension and is likely multifactorial in etiology in those patients. Symptomatic patients had a shorter CART hospitalization time (*p* = 0.035) which is quite counterintuitive as we would have expected a longer CART hospitalization time resulting in more deconditioning to be associated with symptoms. Also, in the univariate analysis, a higher pre-CART ejection fraction was associated with symptoms (*p* = 0.027). This has not been shown in previous studies even in the general population and there is no clear hypothetical mechanism to explain this finding. In addition, in the multivariate analysis pre-CART ejection fraction was not associated with symptoms. History of hypertension was also associated with symptomatic orthostatic hypotension in the univariate (*p* = 0.008) and multivariate analysis (OR: 14.27, CI: 1.75–234.2). Both symptomatic orthostatic hypotension and hypertension can be co-morbid conditions. Orthostatic hypotension has been shown to be more common in elderly patients with hypertension [20]. Only a small percentage of symptomatic orthostatic patients in our study were on anti-hypertensive medications, so the association cannot be merely attributed to medication related side effects. Increased arterial stiffness due to hypertension may play a key role as a previous study has demonstrated that orthostatic patients with falls had a higher arterial wall stiffness than their counterparts [21].

Our study was limited primarily by the small population size. Approximately 20% of patients did not have their orthostatic vital signs assessed which further reduced the sample size. This limited the power of the study and therefore missed associations that we may have uncovered with a larger sample size. Furthermore, few patients in our study population had troponin values assessed which was unfortunate as troponin positivity in CART patients has been shown to be associated with major adverse cardiovascular events [12]. We did not have patients with grade 3 or grade 4 CRS in our study which could have helped us identify a more significant relationship between CRS and orthostasis. 95% of patients in our cohort received YESCARTA which also limits the generalizability of our findings. We are unable to conclude if orthostatic hypotension is a potential side effect across all CART products or more common with certain CAR constructs. Our three month follow-up post CART hospitalization discharge may have missed some patients who developed orthostatic hypotension at a later time. Also, the retrospective aspect does not allow us to ascertain whether findings like higher CRS grade or history of hypertension have any causal relationship to orthostatic hypotension. Further studies in larger cohorts will be needed to be determine if our findings can be generalized to the CART patient population at large.

## Conclusion

There is a high incidence of orthostatic hypotension after CART therapy and orthostatic vital signs should be measured in every patient even after they are discharged from their CART-hospitalization. Similar to the general population, it is more common in older and lower BMI patients. Patients with higher grade CRS may be more likely to have orthostatic hypotension and vital signs should be monitored more closely. Patients with a history of hypertension were more likely to be symptomatic from their orthostatic hypotension. Future directions include replicating these findings in other cohorts and also investigating if orthostatic hypotension after CART therapy results in other outcomes like falls and major adverse cardiovascular events.

## Data Availability

All data generated or analyzed during this study are included in this published article.

## Abbreviations

CART: Chimeric antigen receptor T-cell
CRS: Cytokine release syndrome
ICANS: Immune effector cell-associated neurotoxicity syndrome
ASTCT: American Society for Transplantation and Cellular Therapy
BMI: Body mass index
ALL: Acute Lymphocytic Leukemia
DLBCL: Diffuse Large B-Cell Lymphoma
CHL: Classical Hodgkin Lymphoma
R-CHOP: Rituximab, Cyclophosphamide, Doxorubicin, Vincristine, Prednisone
R-ICE: Rituximab, Ifosfamide, Carboplatin, Etoposide
GEM-OX: Gemcitabine, Oxaliplatin
R-EPOCH: Rituximab, Etoposide, Prednisone, Vincristine, Cyclophosphamide, Doxorubicin
SBP: Systolic blood pressure
DBP: Diastolic blood pressure
HR: Heart rate
HsCRP: High sensitivity C- reactive protein
ACEI: ACE inhibitor
ARB: Angiotensin receptor blocker
CCB: Calcium channel blocker
